# Esthetic perception of orthodontic appliances by Brazilian children and adolescents

**DOI:** 10.1590/2177-6709.21.5.058-066.oar

**Published:** 2016

**Authors:** Deise Caldas Kuhlman, Tatiana Araújo de Lima, Candice Belchior Duplat, Jonas Capelli

**Affiliations:** 1 Specialist in Orthodontics, Universidade do Estado do Rio de Janeiro (UERJ), Rio de Janeiro, Rio de Janeiro, Brazil.; 2 Assistant Professor, Universidade Veiga de Almeida (UVA), Rio de Janeiro, Rio de Janeiro, Brazil. Post-doc resident, Universidade do Estado do Rio de Janeiro (UERJ), Department of Orthodontics, Rio de Janeiro, Rio de Janeiro, Brazil.; 3 MSc, in Orthodontics,Universidade do Estado do Rio de Janeiro (UERJ), Rio de Janeiro, Rio de Janeiro, Brazil.; 4 Associate Professor, Universidade do Estado do Rio de Janeiro (UERJ), Department of Orthodontics, Rio de Janeiro, Rio de Janeiro, Brazil.

**Keywords:** Orthodontic appliances, Esthetics, Corrective orthodontic treatment

## Abstract

**Objective:**

The objective of this present study was to understand how children and adolescents perceive esthetic attractiveness of a variety of orthodontic appliances. It also analyzed preferences according to patients' age, sex and socioeconomic status.

**Methods:**

A photograph album consisting of eight photographs of different orthodontic appliances and clear tray aligners placed in a consenting adult with pleasing smile was used. A sample of children or adolescents aged between 8 and 17 years old (n = 276) was asked to rate each image for its attractiveness on a visual analog scale. Comparisons between the appliances attractiveness were performed by means of nonparametric statistics with Friedman's test followed by Dunn's multiple comparison post-hoc test. Correlation between appliances and individuals' socioeconomic status, age, sex, and esthetic perception was assessed by means of Spearman's correlation analysis.

**Results:**

Attractiveness ratings of orthodontic appliances varied nonsignificantly for children in the following hierarchy: traditional metallic brackets with green elastomeric ligatures > traditional metallic brackets with gray elastomeric ligatures > sapphire esthetic brackets; and for adolescents, as follows: sapphire esthetic brackets > clear aligner without attachments > traditional metallic brackets with green elastomeric ligatures. The correlation between individuals' socioeconomic status and esthetic perception of a given appliance was negative and statistically significant for appliances such as the golden orthodontic brackets and traditional metallic brackets with green elastomeric ligatures.

**Conclusion:**

Metal appliances were considered very attractive, whereas aligners were classified as less attractive by children and adolescents. The correlation between esthetic perception and socioeconomic status revealed that individuals with a higher socioeconomic level judged esthetics as the most attractive attribute. For those with higher economic status, golden orthodontic brackets and traditional metallic brackets with green elastomeric ligatures were assessed as the worst esthetic option.

## RESUMO

**Objetivo:**

o objetivo do presente estudo foi compreender como crianças e adolescentes percebem a atratividade estética de uma variedade de aparelhos ortodônticos. Também foram analisadas as preferências segundo idade, sexo e nível socioeconômico.

**Métodos:**

foi utilizado um álbum de fotografias contendo oito imagens de um mesmo adulto sorrindo, utilizando diferentes aparelhos ortodônticos e alinhadores estéticos. Crianças e adolescentes, com idades entre 8 e 17 anos (n = 276), foram orientadas a classificar cada imagem em uma escala visual analógica, de acordo com a atratividade. Comparações entre a atratividade dos aparelhos foram realizadas por meio do teste estatístico não paramétrico de Friedman seguido pelo teste *post-hoc* de Dunn para comparações múltiplas. As correlações entre a atratividade de cada aparelho e o nível socioeconômico, idade e sexo foram avaliadas segundo o coeficiente de correlação de Spearman.

**Resultados:**

a atratividade dos aparelhos ortodônticos variou, de maneira não significativa, para as crianças na seguinte ordem: braquete metálico com ligadura verde > braquete metálico tradicional com ligadura cinza > braquete estético de safira. Para os adolescentes, variou na seguinte ordem: braquete estético de safira > alinhadores estéticos transparentes sem anexos > braquete metálico com ligadura verde. A correlação entre o nível socioeconômico e a percepção estética foi positiva e estatisticamente significativa para os alinhadores transparentes sem anexos, para os adolescentes do sexo feminino.

**Conclusões:**

os aparelhos metálicos foram considerados muito atrativos pelas crianças, enquanto o braquete estético e os alinhadores transparentes foram classificados como mais atrativos pelos adolescentes. A correlação entre a percepção estética e o nível socioeconômico revelou que o nível socioeconômico não influenciou na atratividade para crianças e adolescentes do sexo masculino. No entanto, adolescentes do sexo feminino com maior nível socioeconômico avaliaram os dispositivos estéticos como os mais atrativos.

## INTRODUCTION

Over the centuries, the concept and meaning of esthetics has changed. In the past, what was considered as appreciation or enjoyment of beauty now includes emotional embellishments, such as judgments of beauty and attractiveness, as well as the psychophysiological arousal patterns associated with it. Therefore, beauty, as an esthetic experience, has been defined as the quality or combination of qualities that provides keen pleasure.^1^

Perception of beauty is not only an individual preference, but it can also be dependent on various cultural, social, geographic, and psychological factors.^2^ ^,^ ^3^ ^,^ ^4^

Orthodontics has greatly evolved regarding esthetic material in response to public demand and available technology, especially with the underlying goal of reducing appliance visibility.^5^ The esthetic paradigm shift in Orthodontics has shown the urgency of incorporating esthetics into the functional goals of orthodontic treatment, leading to an increase in the demand for more inconspicuous orthodontic appliances and more acceptability of orthodontic treatment.^5^ ^,^ ^6^ ^,^ ^7^ ^,^ ^8^ Orthodontic patients and practitioners have now a variety of new treatment options, among which are plastic and ceramic brackets and clear aligners.^9^

A few studies have investigated adult patients' perception of orthodontic appliance esthetics. Those studies reveal that adult consumers value less metal showing in their brackets.^7^ ^,^ ^8^ Esthetic perception and economic value of orthodontic appliances among adults was investigated by Feu et al^10^ who found that sex and age influence perception of orthodontic appliance attractiveness. Walton et al^11^ found a statistically significant difference between sex and age, concluding that American adolescents have a significant preference for metallic brackets.

Considering that age influences the perception of esthetics, understanding the factors involved in the perception of different orthodontic appliances in a particular population enables better planning of resources and strategies in private practice. The appearance of orthodontic appliance plays a significant role in patients' decisions to undergo orthodontic therapy. ^7^

Children and adolescents represent the majority of patients in orthodontic practice, and their perception is increasingly influenced by social media.^12^ Even though orthodontists can use different resources to increase patients' acceptability of orthodontic treatment, a few studies have asked children and adolescents what they favor in terms of orthodontic appliances and esthetics.^1^ ^1^

The aim of this study was to understand not only how children and adolescents perceive esthetic attractiveness, but also their level of acceptance for a variety of orthodontic appliances. In this study, we analyzed preferences according to different ages, sex and socioeconomic status. 

## MATERIAL AND METHODS

This research was approved by Universidade Estadual do Rio de Janeiro (UERJ) Ethics Committee.

To evaluate esthetic attractiveness of orthodontic appliances, we used one photographic sheet with the volunteer's smile shown in eight different situations: (A) with fixed sapphire esthetic brackets, clear elastomeric ligatures (American Orthodontics, Wisconsin, USA) and 0.020-in stainless steel archwire (GAC International, New York, USA); (B) with a clear tray aligner with attachments; (C) with fixed golden orthodontic brackets and clear elastomeric ligatures (American Orthodontics, Wisconsin, USA); (D) with a fixed metallic self-ligating system; (E) with fixed traditional metallic brackets with gray elastomeric ligatures; (F) with fixed sapphire esthetic brackets, clear elastomeric ligatures (American Orthodontics, Wisconsin, USA) and 0.018-in esthetic nickel titanium coated archwire (American Orthodontics, Wisconsin, USA); (G) with a clear tray aligner without attachments; (H) similar to (E), but with green elastomeric ligatures (Morelli, São Paulo, Brazil). 

The volunteer was asked to sign an informed consent form, allowing the digitally captured image of his/her circumoral region to be used. 

**Figure 1:**
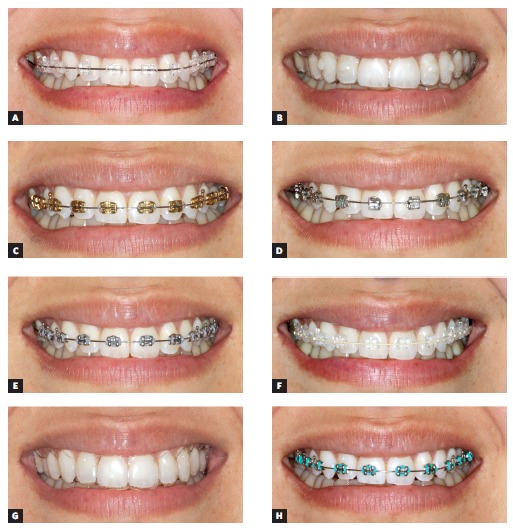
Images used for attractiveness evaluation (photographic sheet): A) sapphire esthetic brackets with stainless steel archwire; B) clear tray aligner with attachments; C) golden orthodontic brackets; D) metallic self-ligating system; E) traditional metallic brackets with gray elastomeric ligatures; F) sapphire esthetic brackets with esthetic coated archwire; G) clear tray aligner without attachments and H) traditional metallic brackets with green elastomeric ligatures.

The sheet was 29.7 cm x 21 cm, and each photograph was 10 cm x 5 cm in size. The eight images, as described above, were randomly grouped in a grid and labeled with letters A to H (Fig 1). For more details on the methods of image acquisition and standardization, readers are referred to the study of Feu et al.^10^

Study participants included any willing child or adolescent aged between 8 and 17 years old who was regularly enrolled in the following schools located in Rio de Janeiro: Instituto de Aplicação Fernando Rodrigues da Silveira (CAP-UERJ), Escola Municipal Republica Argentina, Colégio Maria Rhayte and Instituto Santa Rosa. In addition, in order to participate in the survey, the volunteers presented an informed consent form signed by their parents or a legally responsible adult.

All surveys included demographic and socioeconomic status information forms, instructions, the image-rating scales and the sheet with the photographs. Socioeconomic status was measured based on the "Brazilian Economic Classification Criteria"^13^ that classifies people into eight socioeconomic categories according to the educational level of the household's head and the ownership and consumption of common goods and services within the household.

Each rater (n = 276) received the sheet containing the photograph with the volunteer smiling and a rating sheet with a 100-mm visual analog scale (VAS). The straight lines on the left side of the rating sheet indicated "very unattractive," whereas on the right side the lines stood for "very attractive." The raters were presented with the images (Fig 1) and instructed to rate them based on the VAS. After attractiveness evaluation, the raters were instructed to fill in two different tables, one corresponding to rated items and the other to the level of education of the household head, allowing for classification of socioeconomic status.

The sample was divided into two groups according to age: Group 1 (8-12 years old) and Group 2 (13-17 years old). The median for the total sample raters' age was 11 years old (interquartile range = 10-14); for Group 1, it was 10 years old (interquartile range = 10-11) and for Group 2, it was 15 years old (interquartile range = 14-16). The socioeconomic status in each group is described in Table 1.

**Table 1:**
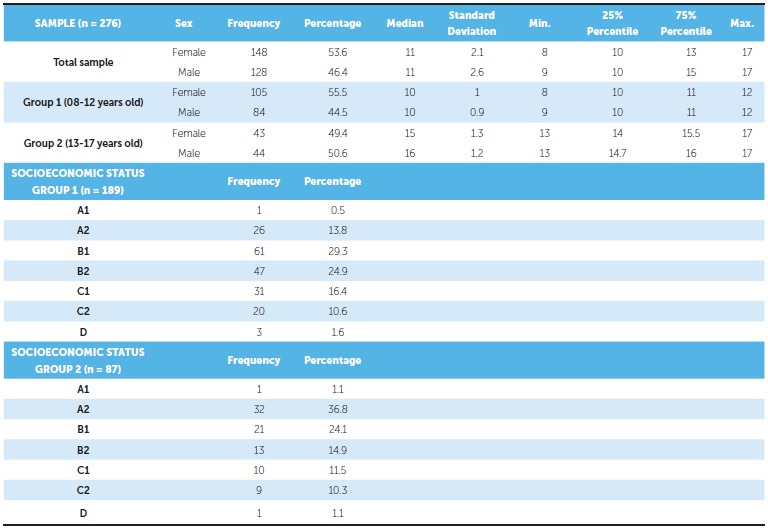
Sample description.

The scores were measured by a previously calibrated dentist using a digital caliper (MGF 505646, Mitutoyo, Tokyo, Japan) positioned on the left-most point of each line of the VAS and which opened to the mark made by the rater. Values were measured in millimeters.

### Statistical analysis

The results from a previous similar study conducted by Rosvall et al^8^ were used for sample size calculation. If the true difference between esthetic perception was 0.030 times the standard deviation, sample size calculation showed that a total of 276 individuals would be needed to provide an 80% probability of the study detecting a esthetic perception difference at a 0.05 significance level. To evaluate intraexaminer reliability, we selected ten surveys randomly and analyzed them twice, with a minimum interval of seven days between each measurement. The values were compared by intraclass correlation coefficient (ICC).

Descriptive statistics for the VAS ratings of perceived attractiveness were calculated. The Kolmogorov-Smirnov test showed lack of normality for distribution and heteroscedasticity for all groups. Comparisons of group attractiveness were carried out by means of nonparametric statistics with Friedman's test followed by Dunn's multiple comparison post-hoc test. The correlation between socioeconomic status and esthetic perception was calculated by means of Spearman correlation analysis and was represented with the "r" value. Raters' age was segmented to assess whether it could influence the results. 

## RESULTS 

Intraexaminer reliability was high for attractiveness assessment (mean ICC = 0.98; 95% CI = 0.98-0.99), thus indicating substantial consistency. 

Descriptive statistics for VAS ratings of perceived attractiveness for each group are reported in Table 2. Higher VAS scores (0-100) suggest greater appliance attractiveness. Traditional metallic brackets with green elastomeric ligatures had the best evaluation scores, followed by traditional metallic brackets with gray elastomeric ligatures in Group 1. However, clear tray aligners with attachments had the worst evaluation preceded by clear tray aligners without attachments. In Group 2, sapphire brackets with esthetic archwire presented the highest score, followed by clear tray aligners without attachments. 

**Table 2:** Statistical comparison of VAS ratings representing attractiveness evaluation with Friedman's (*p* < 0.001) and Dunn's post-hoc test by age.

GROUP 1 - 08-12 years (n = 189)			
VAS	Median	25% Percentile	75% Percentile
A) Sapphire brackets with metallic archwire	37^a^	19	61
B) Clear aligner with attachments	14^b^	6	29
C) Golden metallic brackets	50^a,c^	18	77
D) Self-ligated metallic brackets	38^a,c^	20	61
E) Metallic brackets with gray ties	52^a,c^	30	72
F) Sapphire brackets with esthetic archwire	50^c^	32	81
G) Clear aligner without attachments	17^b^	7	38
H) Metallic brackets with green ties	75	50	92
GROUP 2 - 13-17 years (n = 87)			
VAS	Median	25% Percentile	75% Percentile
A) Sapphire brackets with metallic archwire	46^a,b^	25	78
B) Clear aligner with attachments	24^a,c^	9	63
C) Golden metallic brackets	16^c^	6	45
D) Self-ligated metallic brackets	32^a,c^	13	54
E) Metallic brackets with gray ties	43^a,b^	28	72
F) Sapphire brackets with esthetic archwire	59^b^	33	83
G) Clear aligner without attachments	47^a,b^	11	74
H) Metallic brackets with green ties	46^a,b^	19	83

Distinct superscripts letters indicate statistical significance.

The perceived attractiveness of appliances for males and females was analyzed for each group (Table 3 and 4). Males tended to assign lower scores for some types of appliances. In Group 1, females rated the most attractive appliance as follows: fixed traditional metallic brackets with green elastomeric ligatures; and fixed traditional metallic brackets with gray elastomeric ligatures (a statistically significant difference was noted between them). Males better-rated appliances were as follows: fixed traditional metallic brackets with green elastomeric ligatures; and fixed golden orthodontic brackets, with differences among the two brackets. The worst rating for both males and females were the clear tray aligners with and without attachments. However, the most attractive appliances for females in Group 2 were as follows: sapphire brackets with esthetic archwire; fixed traditional metallic brackets with green elastomeric ligatures; and fixed sapphire brackets with metallic archwire (the first had no difference among the second and the third brackets). For males, clear tray aligners without attachments; sapphire esthetic brackets with esthetic archwire; and traditional metallic brackets with gray elastomeric ligatures had the best evaluation scores, without differences among the appliances.

**Table 3:** Statistical comparison of VAS ratings representing attractiveness evaluation with Friedman's (*p* < 0.001) and Dunn's post-hoc test by sex for Group 1.

GROUP 1 - 08-12 years (n= 189)			
FEMALE			
VAS	Median	25% Percentile	75% Percentile
A) Sapphire brackets with metallic archwire	38^a,b^	18	61
B) Clear aligner with attachments	13^c^	6	27
C) Golden metallic brackets	35^a^	16	66
D) Self-ligated metallic brackets	38^a^	20	60
E) Metallic brackets with gray ties	56^a^	28	80
F) Sapphire brackets with esthetic archwire	55^a,d^	31	82
G) Clear aligner without attachments	18^b,c^	7	47
H) Metallic brackets with green ties	81^d^	60	94
MALE			
VAS	Median	25% Percentile	75% Percentile
A) Sapphire brackets with metallic archwire	35^a,b^	19	64
B) Clear aligner with attachments	15^a,c^	7	33
C) Golden metallic brackets	62^c^	26	82
D) Self-ligated metallic brackets	42^a,c^	19	66
E) Metallic brackets with gray ties	48^a,b^	32	67
F) Sapphire brackets with esthetic archwire	42^b^	32	75
G) Clear aligner without attachments	15^a,b^	7	28
H) Metallic brackets with green ties	68^a,b^	44	89

Distinct superscripts letters indicate statistical significance.

**Table 4:** Statistical comparison of VAS ratings representing attractiveness evaluation with Friedman's (*p* < 0.001) and Dunn's post-hoc test by sex for Group 2.

GROUP 2 - 13-17 years (n= 87)			
FEMALE			
VAS	Median	25% Percentile	75% Percentile
A) Sapphire brackets with metallic archwire	56^a^	29	78
B) Clear aligner with attachments	16^b^	6	63
C) Golden metallic brackets	16^a^	8	42
D) Self-ligated metallic brackets	29^a^	11	43
E) Metallic brackets with gray ties	40^a^	27	71
F) Sapphire brackets with esthetic archwire	64^a,c^	39	84
G) Clear aligner without attachments	55^b^	21	86
H) Metallic brackets with green ties	56^c^	19	88
MALE			
VAS	Median	25% Percentile	75% Percentile
A) Sapphire brackets with metallic archwire	40^a^	22	82
B) Clear aligner with attachments	31^a,b^	11	64
C) Golden metallic brackets	15^b^	5	59
D) Self-ligated metallic brackets	37^a,b^	15	64
E) Metallic brackets with gray ties	45^a^	29	74
F) Sapphire brackets with esthetic archwire	52^a^	29	78
G) Clear aligner without attachments	70^a,b^	42	70
H) Metallic brackets with green ties	34^a,b^	16	80

Distinct superscripts letters indicate statistical significance.

The correlation between socioeconomic status and esthetic attractiveness in Group 1 was weak for all orthodontic appliances, considering both females and males, as seen in Table 5. On the other hand, for Group 2, females showed a moderate positive correlation with clear tray aligner without attachments (option G) and a moderate negative correlation with metallic brackets with green ties (option H), as seen in Table 5.

**Table 5:** Spearman rank correlation coefficients (r) between socioeconomic status and attributed value by sex for each group.

GROUP 1 - 08-12 years (n = 189)	Female	Male
A) Sapphire brackets with metallic archwire	-0.146	0.102
B) Clear aligner with attachments	-0.024	-0.066
C) Golden metallic brackets	-0.063	-0.078
D) Self-ligated metallic brackets	0.103	0.067
E) Metallic brackets with gray ties	0.022	0.161
F) Sapphire brackets with esthetic archwire	-0.011	-0.080
G) Clear aligner without attachments	-0.013	-0.034
H) Metallic brackets with green ties	0.133	-0.005
GROUP 2 - 13-17 years (n = 87)	Female	Male
A) Sapphire brackets with metallic archwire	0.201	0.113
B) Clear aligner with attachments	0.273	-0.036
C) Golden metallic brackets	-0.293	-0.229
D) Self-ligated metallic brackets	0.062	-0.130
E) Metallic brackets with gray ties	-0.206	-0.180
F) Sapphire brackets with esthetic archwire	-0.237	0.263
G) Clear aligner without attachments	0.364*	0.292
H) Metallic brackets with green ties	-0.400	0.198

*p < 0.05.

## DISCUSSION

Regarding the type of brackets, children, adolescents and adults may present different preferences, and appliance attractiveness can be used to facilitate patients' acceptance.^11^

Among the various designs available, the orthodontist should provide appliances that are acceptable to patients and also work in harmony with the appliance biomechanics.^6^ In this sense, it may be reassuring to know that traditional metallic brackets with colored (i.e., green) elastomeric ligatures were rated as more attractive by children.

The perception of attractiveness was influenced by age. Group 2 (13-17 years old) showed greater preference for more esthetic appliances than Group 1 (8-12 years old). Younger patients evaluated traditional metallic brackets with green elastomeric ligatures more positively, with higher scores than sapphire brackets. This study found that traditional metallic brackets with green elastomeric ligatures are preferred among children (Group 1), followed by traditional metallic brackets with gray elastomeric ligatures and sapphire esthetic brackets with esthetic coated archwire, with no statistically significant difference among them. Clear tray aligners with and without attachments had the worst esthetic perception for this group. These data show that reduction of visible metal is not a determining esthetic factor for most children. A similar result was found in the study by Walton et al,^11^ but their result was different from similar studies in adults, which found preferences for appliances with less visible metal.^7^ ^,^ ^8^ ^,^ ^10^ ^,^ ^14^ Group 2 gave better scores for sapphire brackets and clear aligner without attachments than Group 1, and these findings were similar to results yielded by Walton et al^11^ and Oliveira et al.^14^

Regarding individuals' sex, in Group 1, there was a strong preference for traditional metallic brackets with green elastomeric ligatures by both males and females, although there was no statistically significant difference when compared to other appliances. On the other hand, clear tray aligners received the worst scores among females and males. These appliances achieved similar scores of attractiveness from both males and females in Group 1, and expressed a non-statistically significant difference when attachments were present. In the present study, sex was not an influential factor in orthodontic appliance attractiveness for children. In Group 2, females gave higher scores for attractiveness to the following appliances: sapphire esthetic brackets with esthetic coated archwire, traditional metallic brackets with green elastomeric ligatures and sapphire esthetic brackets with stainless steel archwire. For male individuals, there was no difference in attractiveness among clear tray aligner without attachments, sapphire esthetic brackets with stainless steel archwire and traditional metallic brackets with gray elastomeric ligatures. In this sense, these results were different from Walton et al^11^ and Feu et al^10^ who found differences in the esthetic perception of orthodontic appliances among males and females. Males and females rated golden orthodontic brackets as less attractive. 

The results of this study suggest that age and sex influence differently the perception of attractiveness. Adolescents tended to have a stronger preference for clear appliances than children. In that perspective, male adolescents showed attractiveness for more esthetic orthodontic appliances, while no difference was found among female adolescents when comparing attractiveness between the two better-rated appliances: one esthetic (sapphire esthetic brackets with stainless steel archwire) and another non-esthetic (traditional metallic brackets with green elastomeric ligatures). In fact, the present study, as well as other two studies,^10^ ^,^ ^11^ have shown similar results about esthetic perception of orthodontic appliances between males and females at different ages.

The correlation between socioeconomic status of users and esthetic perception of an appliance in Group 1 was weak and non-statistically different for all orthodontic appliances, and according to both males and females. The same result applies to Group 2 for males. These results suggest that one's socioeconomic status does not influence attractiveness for children and male adolescents. The scores attributed by females in Group 2 presented a positive moderate statistically significant correlation with the clear aligner without attachments, and a negative moderate non-statistically significant correlation with metallic brackets combined with green elastomeric ligatures. Because the correlation of the esthetic pattern was positive and significant, the higher the socioeconomic level, the higher the preference for this alternative in female adolescents. These findings corroborate those yielded by Feu et al^10^ and Rosvall et al^8^ who found that adults who had a higher socioeconomic level would pay more for more esthetic options, such as sapphire esthetic brackets, lingual brackets or clear tray aligners. When considering this finding in other socioeconomic realities, different results could be found in different Brazilian samples; therefore, further studies are needed in this area. Therefore, the results of the present study cannot be applied indiscriminately to all socioeconomic groups. The importance of these aspects must be studied and known in each type of analysis.

## CONCLUSIONS

Orthodontic appliance attractiveness according to group varies non-significantly for children as follows: traditional metallic brackets with green elastomeric ligatures > traditional metallic brackets with gray elastomeric ligatures > sapphire esthetic brackets; and for adolescents, as follows: sapphire esthetic brackets > clear aligner without attachments > traditional metallic brackets with green elastomeric ligatures.

Metal appliances, widely used in clinical practice, were considered very attractive, although aligners, which are seen as the most esthetic option, were classified as less attractive for male and female children. With regard to adolescents, males showed a preference for esthetic appliances while females presented no difference between an esthetic and a metallic appliance. However, when the correlation between esthetic perception and socioeconomic status was made, it was observed that individuals with a higher socioeconomic level judged esthetics as the most attractive attribute for female adolescents. For those with a higher economic status, traditional metallic brackets with green elastomeric ligatures were assessed as the worst esthetic option.
